# Supplementary figures and images for: Selective retention of dysfunctional mitochondria during asymmetric cell division in yeast
Source: PLoS Biol. 2023 Sep 18;21(9):e3002310. doi: 10.1371/journal.pbio.3002310 (PMC10538663; doi:10.1371/journal.pbio.3002310)

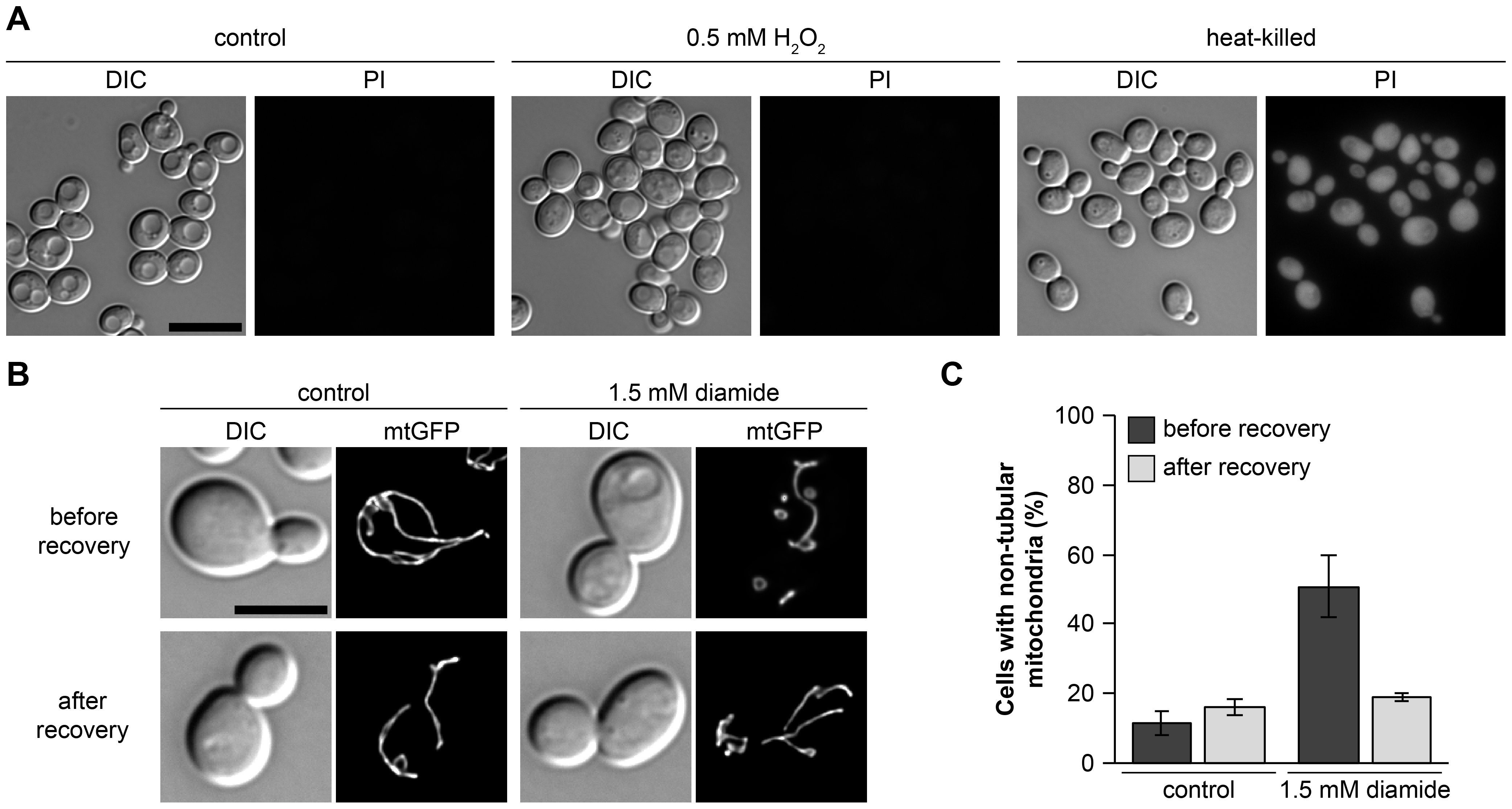

Supplement: S1 Fig — (A) Yeast cells were treated with 0.5 mM H2O2 for 15 min, stained with PI, and analyzed by DIC and fluorescence microscopy. Cells that were killed at 100°C served as a positive control. Images were taken with identical camera settings. Bar, 10 μm. A quantification is shown in Fig 1C. (B and C) Yeast cells expressing mtGFP were treated with exogenously added diamide for 30 min and analyzed by DIC and fluorescence microscopy. For recovery cells were incubated for 90 min in fresh medium. Fluorescence images are z stacks subjected to deconvolution. Mitochondrial morphology was quantified in 150 cells per sample (triplicate experiments ± SD). Bar, 5 μm. The data underlying this figure can be found in S1 Datasheet. (TIF) [file pbio.3002310.s001.tif]

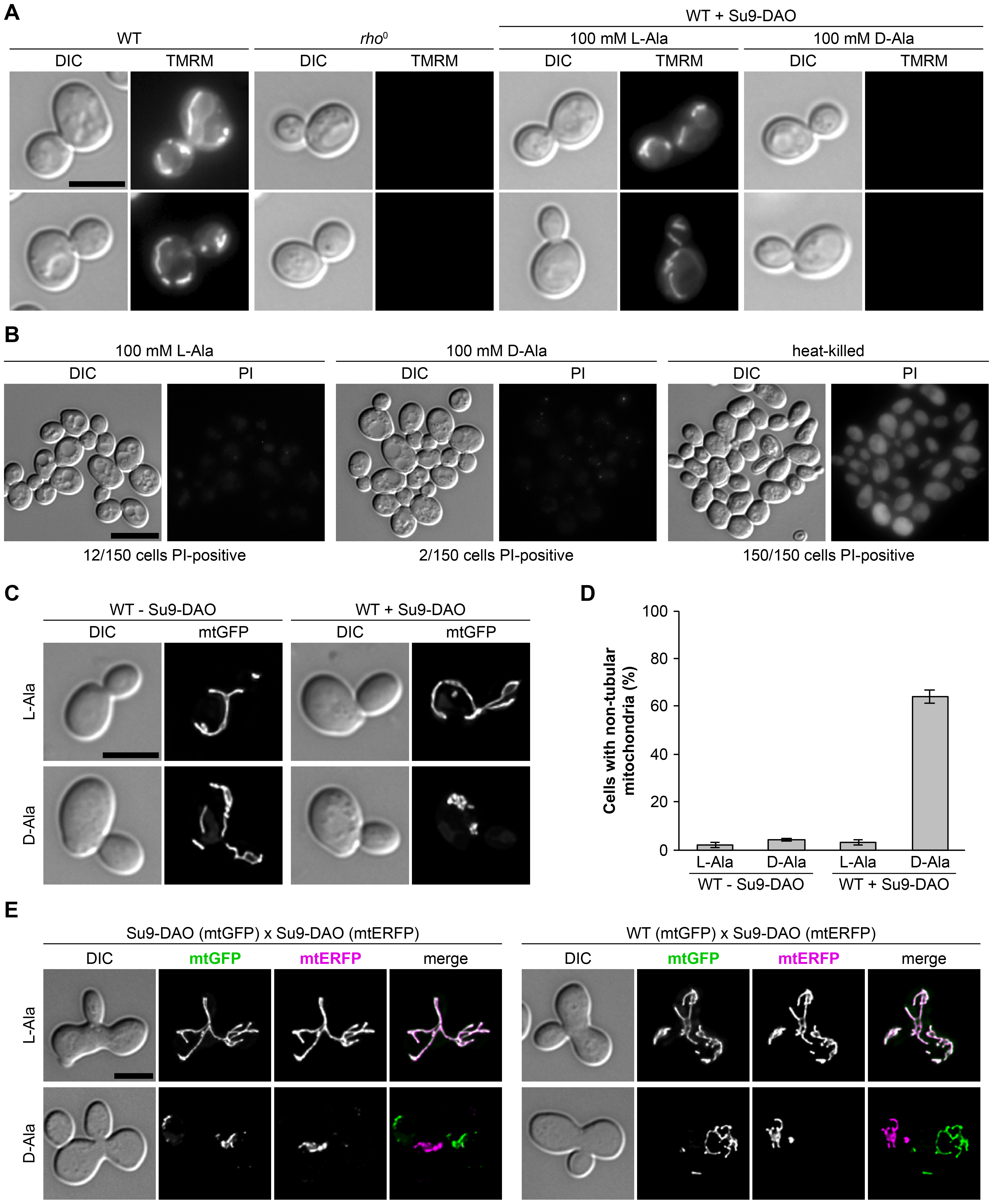

Supplement: S2 Fig — (A) Wild-type cells, cells lacking mtDNA (rho0), and wild-type cells expressing Su9-DAO were incubated without or with L-alanine or D-alanine for 3 h and then stained with TMRM. Fluorescence microscopy images are maximum intensity projections of z stacks using identical camera settings. Bar, 5 μm. (B) Wild-type cells expressing Su9-DAO were incubated in the presence of L-alanine or D-alanine for 3 h and stained with PI. Heat-killed cells were incubated for 5 min at 100°C. Fluorescence images were taken with identical camera settings. Bar, 10 μm. (C and D) Wild-type cells expressing mtGFP and lacking (left) or expressing (right) Su9-DAO were incubated for 3 h in the presence of L-alanine or D-alanine. Fluorescence images are z stacks subjected to deconvolution. Mitochondrial morphology was quantified in 100 cells per sample (3 biological replicates ± SD). Bar, 5 μm. (E) Wild-type cells expressing either Su9-DAO together with mtGFP (left) or only mtGFP (right) and wild-type cells expressing Su9-DAO together with mtERFP were incubated for 30 min with L-alanine or D-alanine, mixed, and incubated for another 3 h to allow mating and zygote formation. Fluorescence images are z stacks subjected to deconvolution. Single channel images shown here were used to generate merged images shown in Fig 2H. Bar, 5 μm. The data underlying this figure can be found in S1 Datasheet. (TIF) [file pbio.3002310.s002.tif]

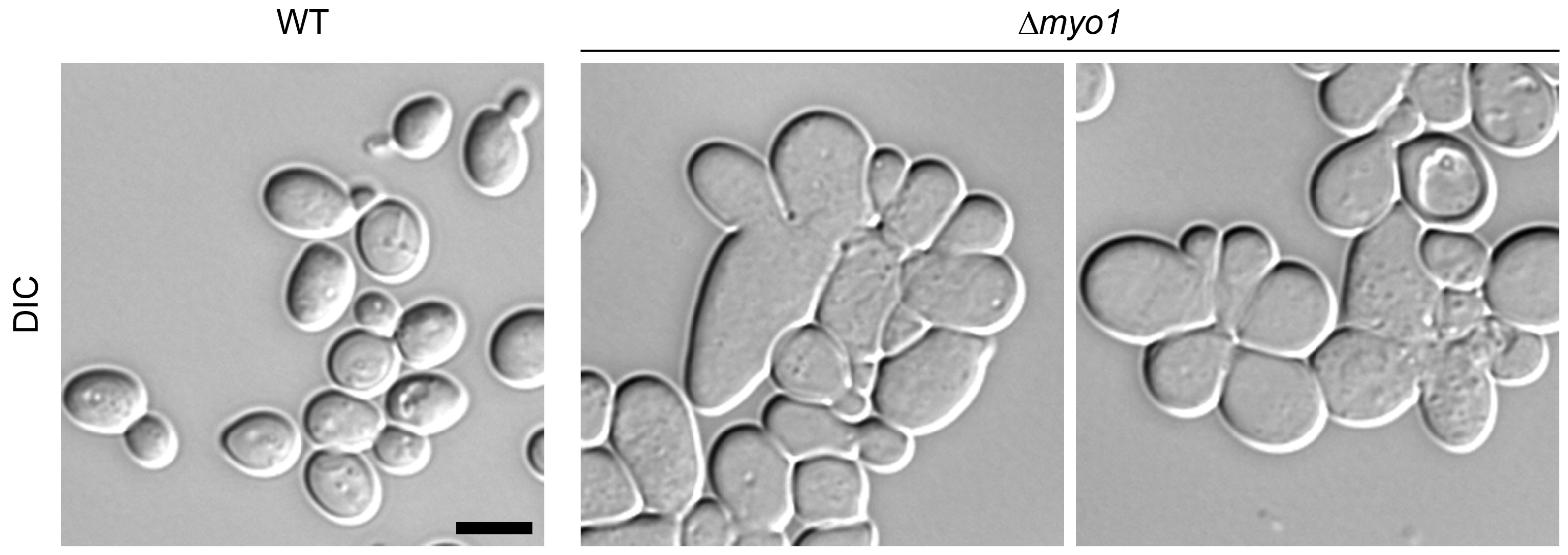

Supplement: S3 Fig — Logarithmically growing cells in minimal complete medium containing glucose as carbon source were analyzed by DIC microscopy. Bar, 5 μm. (TIF) [file pbio.3002310.s003.tif]

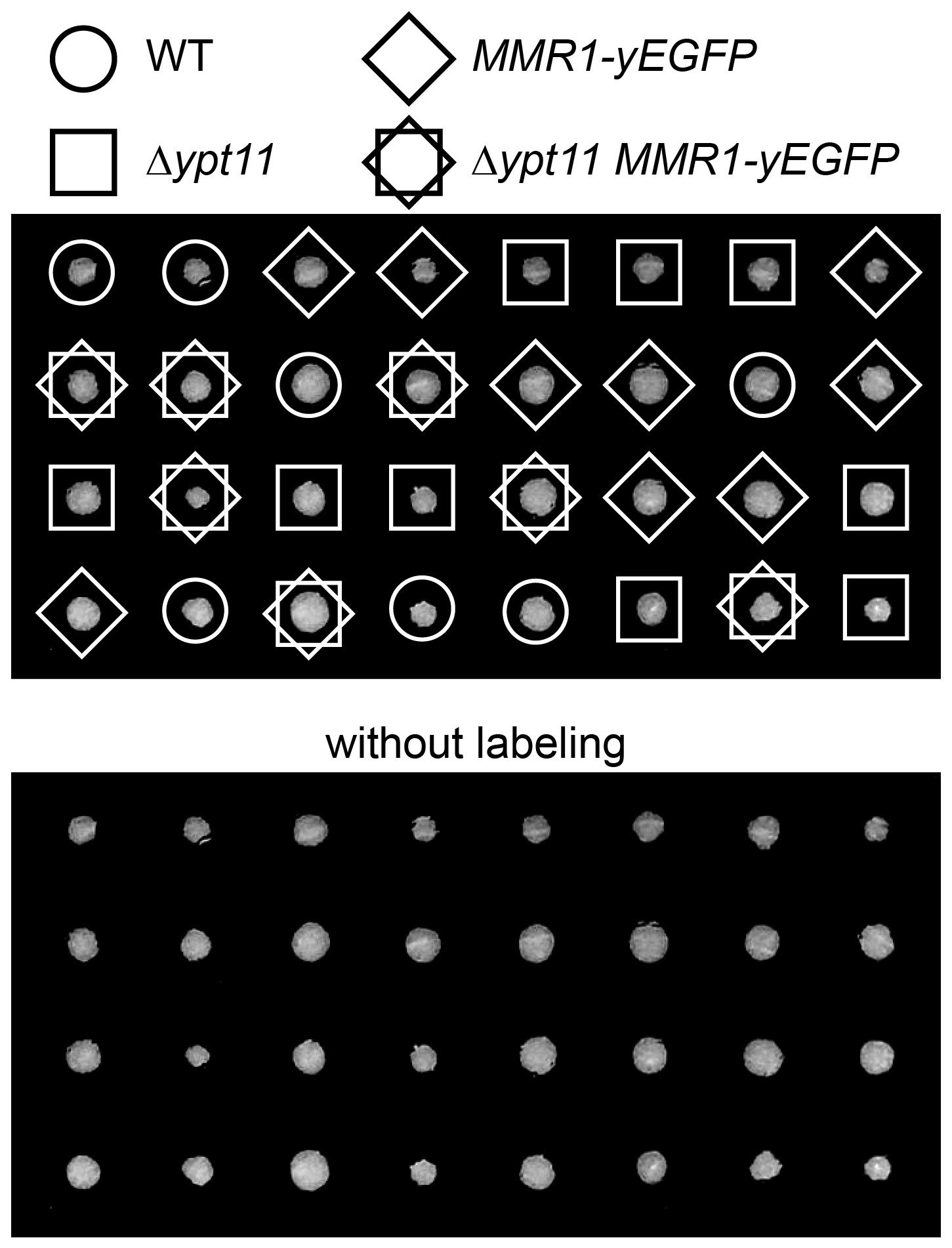

Supplement: S4 Fig — Strain Δypt11 (strain no. 19 in S1 Table) and a wild-type strain carrying a genomic insertion of the MMR1-yEGFP allele at the MMR1 locus (strain no. 15 in S1 Table) were mated, and the resulting diploid strain was subjected to sporulation and tetrad dissection. Ascospores were allowed to grow to colonies on YPD plates. Four colonies in each column correspond to 1 tetrad. Note that all 7 clones carrying both the Δypt11 and MMR1-yEGFP alleles grow like wild type. Compare Fig 7D for the lethal phenotype of Δmmr1 Δypt11 in the genetic background used in this study. (TIF) [file pbio.3002310.s004.tif]

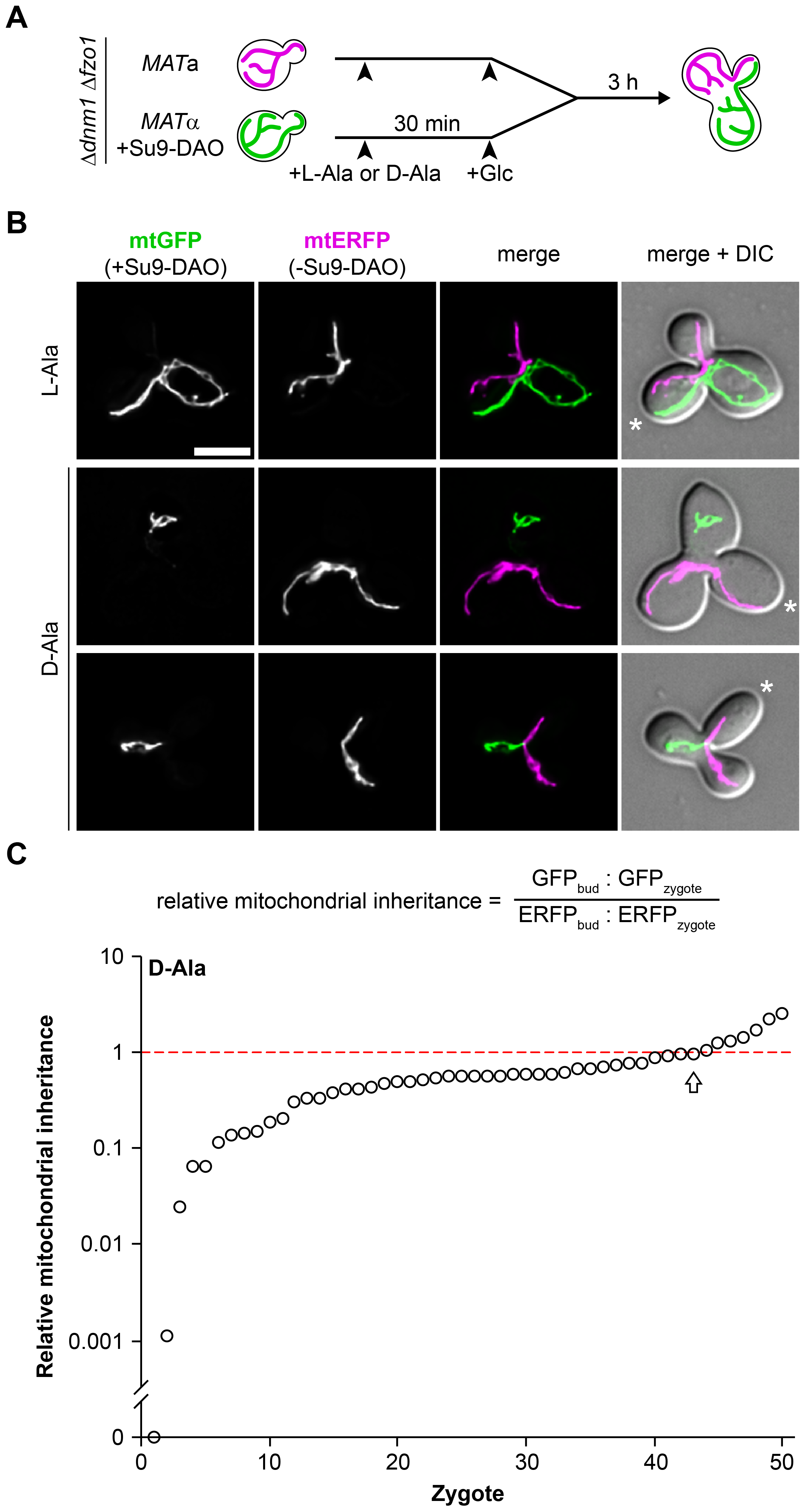

Supplement: S5 Fig — (A) The experiment was performed as in Fig 8, but with switched mating types. (B) Fluorescence images are z stacks subjected to deconvolution. Asterisks indicate medial buds of zygotes. Bar, 5 μm. (C) Relative mitochondrial inheritance was quantified in 50 D-alanine-treated zygotes by calculating the GFP and ERFP intensity ratios as shown in the equation. The arrow points to the zygote that is closest to the value of 1. (TIF) [file pbio.3002310.s005.tif]
