# Supplementary material for: Selective retention of dysfunctional mitochondria during asymmetric cell division in yeast
Source: PLoS Biol. 2023 Sep 18;21(9):e3002310. doi: 10.1371/journal.pbio.3002310 (PMC10538663; doi:10.1371/journal.pbio.3002310)
Supplement: S1 Table — (PDF) [file pbio.3002310.s015.pdf]

**S1 Table. Yeast strains used in this study.**

Plasmids transformed into yeast strains are described in the text and are not indicated in this table.

| No. | Strain | Genotype | Source |
|---|---|---|---|
| 1 | BY4741 | *MAT*a *his3Δ1 leu2Δ0 met15Δ0 ura3Δ0* | [1] |
| 2 | BY4742 | *MAT*α *his3Δ1 leu2Δ0 lys2Δ0 ura3Δ0* | [1] |
| 3 | WT Su9-DAO | *MAT*a *his3Δ1 leu2Δ0 met15Δ0 ura3Δ0 ho::Su9-DAO-natMX6* | this study |
| 4 | WT Su9-DAO | *MAT*α *his3Δ1 leu2Δ0 lys2Δ0 ura3Δ0 ho::Su9-DAO-natMX6* | this study |
| 5 | Myo2-GFP | *MAT*a *his3Δ1 leu2Δ0 met15Δ0 ura3Δ0 MYO2-yEGFP::CaURA3* | this study |
| 6 | Myo2-GFP Su9-DAO | *MAT*a *his3Δ1 leu2Δ0 met15Δ0 ura3Δ0 MYO2-yEGFP::CaURA3 ho::Su9-DAO-natMX6* | this study |
| 7 | Δ*mip1* Su9-DAO | *MAT*a *his3Δ1 leu2Δ0 met15Δ0 ura3Δ0 mip1::kanMX4 ho::Su9-DAO-natMX6* [*rho*$^0$] | this study |
| 8 | Δ*dnm1* Su9-DAO | *MAT*a *his3Δ1 leu2Δ0 met15Δ0 ura3Δ0 dnm1::kanMX4 ho::Su9-DAO-natMX6* | this study |
| 9 | Δ*dnm1* Δ*fzo1* | *MAT*a *his3Δ1 leu2Δ0 lys2Δ0 ura3Δ0 fzo1::kanMX4 dnm1::kanMX4* | this study |
| 10 | Δ*dnm1* Δ*fzo1* | *MAT*α *his3Δ1 leu2Δ0 lys2Δ0 ura3Δ0 fzo1::kanMX4 dnm1::kanMX4* | this study |
| 11 | Δ*dnm1* Δ*fzo1* Su9-DAO | *MAT*a *his3Δ1 leu2Δ0 lys2Δ0 ura3Δ0 fzo1::kanMX4 dnm1::kanMX4 ho::Su9-DAO-natMX6* | this study |
| 12 | Δ*dnm1* Δ*fzo1* Su9-DAO | *MAT*α *his3Δ1 leu2Δ0 lys2Δ0 ura3Δ0 fzo1::kanMX4 dnm1::kanMX4 ho::Su9-DAO-natMX6* | this study |
| 13 | Δ*tpm2* Su9-DAO | *MAT*a *his3Δ1 leu2Δ0 met15Δ0 ura3Δ0 tpm2::kanMX4 ho::Su9-DAO-natMX6* | this study |
| 14 | Δ*myo1* Su9-DAO | *MAT*a *his3Δ1 leu2Δ0 lys2Δ0 ura3Δ0 myo1::kanMX4 ho::Su9-DAO-natMX6* | this study |
| 15 | Mmr1-GFP | *MAT*a *his3Δ1 leu2Δ0 met15Δ0 ura3Δ0 MMR1-yEGFP::CaURA3* | this study |
| 16 | Mmr1-GFP Su9-DAO | *MAT*a *his3Δ1 leu2Δ0 met15Δ0 ura3Δ0 ho::Su9-DAO-natMX6 MMR1-yEGFP::CaURA3* | this study |
| 17 | Δ*mmr1* Su9-DAO | *MAT*a *his3Δ1 leu2Δ0 ura3Δ0 ho::Su9-DAO-natMX6 mmr1::kanMX4* | this study |
| 18 | Δ*mmr1* Su9-DAO | *MAT*a *his3Δ1 leu2Δ0 lys2Δ0 ura3Δ0 ho::Su9-DAO-natMX6 mmr1::kanMX4* | this study |
| 19 | Δ*ypt11* | *MAT*α *his3Δ1 leu2Δ0 lys2Δ0 ura3Δ0 ypt11::HIS3MX6* | this study |
| 20 | Δ*ypt11* Su9-DAO | *MAT*a *his3Δ1 leu2Δ0 met15Δ0 ura3Δ0 ho::Su9-DAO-natMX6 ypt11::kanMX4* | this study |
| 21 | Δ*ypt11* Su9-DAO | *MAT*α *his3Δ1 leu2Δ0 ura3Δ0 ho::Su9-DAO-natMX6 ypt11::HIS3MX6* | this study |
| 22 | Δ*mmr1* Δ*ypt11* Su9-DAO [Tom20-Inp2-GFP] | *MAT*a *his3Δ1 leu2Δ0 ura3Δ0 ho::Su9-DAO-natMX6 mmr1::kanMX4 ypt11::HIS3MX6* [pRS416-Tom20-Inp2-GFP] | this study |
| 23 | Δ*mmr1* Δ*ypt11* Su9-DAO [Tom20-Inp2-GFP] | *MAT*α *his3Δ1 leu2Δ0 lys2Δ0 ura3Δ0 ho::Su9-DAO-natMX6 mmr1::kanMX4 ypt11::HIS3MX6* [pRS416-Tom20-Inp2-GFP] | this study |

| No. | Strain | Genotype | Source |
|---|---|---|---|
| 24 | Δ*mmr1* Su9-DAO | *MAT*a *his3Δ1 leu2Δ0 lys2Δ0 met15Δ0 ura3Δ0 ho::Su9-DAO-natMX6 mmr1::kanMX4* | this study |
| 25 | Δ*dnm1* Δ*fzo1 pGAL* Su9-DAO | *MAT*a *his3Δ1 leu2Δ0 lys2Δ0 ura3Δ0 fzo1::kanMX4 dnm1::kanMX4 ho::pGAL-Su9-DAO-natMX6* | this study |
| 26 | Δ*dnm1* Δ*fzo1* Δ*mmr1* | *MATα his3Δ1 leu2Δ0 lys2Δ0 ura3Δ0 fzo1::kanMX4 dnm1::kanMX4 mmr1::hphNT1* | this study |
| 27 | Δ*dnm1* Δ*fzo1* Δ*mmr1 pGAL* Su9-DAO | *MAT*a *his3Δ1 leu2Δ0 lys2Δ0 ura3Δ0 fzo1::kanMX dnm1::kanMX4 mmr1::hphNT1 ho::pGAL-Su9-DAO-natMX6* | this study |
| 28 | Δ*dnm1* Δ*fzo1* Δ*ypt11* | *MATα his3Δ1 leu2Δ0 lys2Δ0 ura3Δ0 fzo1::kanMX4 dnm1::kanMX4 ypt11::hphNT1* | this study |
| 29 | Δ*dnm1* Δ*fzo1* Δ*ypt11 pGAL* Su9-DAO | *MAT*a *his3Δ1 leu2Δ0 lys2Δ0 ura3Δ0 fzo1::kanMX dnm1::kanMX4 ypt11::hphNT1 ho::pGAL-Su9-DAO-natMX6* | this study |

**Reference**


1. Brachmann CB, Davies A, Cost GJ, Caputo E, Li J, Hieter P, et al. Designer deletion strains derived from *Saccharomyces cerevisiae* S288C: a useful set of strains and plasmids for PCR-mediated gene disruption and other applications. Yeast. 1998;14:115-32. doi: 10.1002/(SICI)1097-0061(19980130)14:2<115::AID-YEA204>3.0.CO;2-2.
