# Supplementary material for: Selective retention of dysfunctional mitochondria during asymmetric cell division in yeast
Source: PLoS Biol. 2023 Sep 18;21(9):e3002310. doi: 10.1371/journal.pbio.3002310 (PMC10538663; doi:10.1371/journal.pbio.3002310)
Supplement: S2 Table — (PDF) [file pbio.3002310.s016.pdf]

**S2 Table. Data pooling and statistics.**

| Fig | Data | Error bars | Statistics |
|---|---|---|---|
| Fig 1B | Mean values of three biological replicates (n=3) based on three independent experiments (N=3); for each replicate 150 cells were evaluated. | Standard deviation | None |
| Fig 1C | Mean values of three biological replicates (n=3) based on three independent experiments (N=3); for each replicate 200 cells were evaluated. | Standard deviation | None |
| Fig 1E | Mean values of three biological replicates (n=3) based on three independent experiments (N=3); for each replicate at least 100 cells were evaluated. | Standard deviation | Unpaired two-tailed Student's t-test with ad hoc normality (Shapiro-Wilk) and equal variance (Brown-Forsythe) tests using mean values of biological replicates collected in independent experiments |
| Fig 1G | Mean values of three biological replicates (n=3) based on three independent experiments (N=3); for each replicate at least 100 cells were evaluated. | Standard deviation | Unpaired two-tailed Student's t-test with ad hoc normality (Shapiro-Wilk) and equal variance (Brown-Forsythe) tests using mean values of biological replicates collected in independent experiments |
| Fig 2D | Mean values of three biological replicates (n=3) based on three independent experiments (N=3); for each replicate 100 cells were evaluated. | Standard deviation | Unpaired two-tailed Student's t-test with ad hoc normality (Shapiro-Wilk) and equal variance (Brown-Forsythe) tests using mean values of biological replicates collected in independent experiments |
| Fig 2F | Mean values of three biological replicates (n=3) based on one independent experiment (N=1); for each replicate 100 cells were evaluated. | Standard deviation | One way analysis of variance (One Way ANOVA) using the biological replicates with ad hoc normality (Shapiro-Wilk) and equal variance (Brown-Forsythe) tests, and post hoc Holm-Sidak test with all pairwise multiple comparisons |
| Fig 2I | Mean values of three biological replicates (n=3) based on three independent experiments (N=3); for each replicate 50 zygotes were evaluated. | Standard deviation | One way analysis of variance (One Way ANOVA) using values of biological replicates collected in independent experiments with ad hoc normality (Shapiro-Wilk) and equal variance (Brown-Forsythe) tests, and post hoc Holm-Sidak test with all pairwise multiple comparisons |
| Fig 3B | Mean values of three biological replicates (n=3) based on three independent experiments (N=3); for each replicate at least 100 cells were evaluated. | Standard deviation | Unpaired two-tailed Student's t-test with ad hoc normality (Shapiro-Wilk) and equal variance (Brown-Forsythe) tests using mean values of biological replicates collected in independent experiments |
| Fig 3D | Mean values of three biological replicates (n=3) based on three independent experiments (N=3); for each replicate 100 cells were evaluated. | Standard deviation | Unpaired two-tailed Student's t-test with ad hoc normality (Shapiro-Wilk) and equal variance (Brown-Forsythe) tests using mean values of biological replicates collected in independent experiments |

| Fig | Data | Error bars | Statistics |
|---|---|---|---|
| Fig 3F | Mean values of three biological replicates (n=3) based on three independent experiments (N=3); for each replicate at least 100 cells were evaluated. | Standard deviation | Unpaired two-tailed Welch's t-test with ad hoc normality (Shapiro-Wilk) and equal variance (Brown-Forsythe) tests using mean values of biological replicates collected in independent experiments |
| Fig 3H | Mean values of three biological replicates (n=3) based on three independent experiments (N=3); for each replicate at least 100 cells were evaluated. | Standard deviation | Unpaired two-tailed Student's t-test with ad hoc normality (Shapiro-Wilk) and equal variance (Brown-Forsythe) tests using mean values of biological replicates collected in independent experiments |
| Fig 4B | Mean values of three biological replicates (n=3) based on three independent experiments (N=3); for each replicate 100 cells were evaluated. | Standard deviation | One way analysis of variance (One Way ANOVA) using mean values of biological replicates collected in independent experiments with ad hoc normality (Shapiro-Wilk) and equal variance (Brown-Forsythe) tests, and post hoc Holm-Sidak test with all pairwise multiple comparisons |
| Fig 4E | Mean values of three biological replicates (n=3) for WT and five biological replicates for Δ*tmp2* (n=5) based on three independent experiments (N=3), for each replicate at least 100 cells were evaluated. | Standard deviation | One way analysis of variance (One Way ANOVA) using values of biological replicates collected in independent experiments with ad hoc normality (Shapiro-Wilk) and equal variance (Brown-Forsythe) tests, and post hoc Holm-Sidak test with all pairwise multiple comparisons |
| Fig 5B | Mean values of three biological replicates (n=3) based on three independent experiments (N=3); for each replicate 150 cells were evaluated. | Standard deviation | One way analysis of variance (One Way ANOVA) using mean values of biological replicates collected in independent experiments with ad hoc normality (Shapiro-Wilk) and equal variance (Brown-Forsythe) tests, and post hoc Holm-Sidak test with all pairwise multiple comparisons |
| Fig 5D | Mean values of three biological replicates (n=3) based on three independent experiments (N=3); for each replicate at least 100 cells were evaluated. | Standard deviation | One way analysis of variance (One Way ANOVA) using mean values of mitochondrial inheritance of biological replicates collected in independent experiments with ad hoc normality (Shapiro-Wilk) and equal variance (Brown-Forsythe) tests, and post hoc Holm-Sidak test with all pairwise multiple comparisons |

| Fig | Data | Error bars | Statistics |
|---|---|---|---|
| Fig 5F | Mean values of three biological replicates (n=3) based on three independent experiments (N=3); for each replicate at least 100 cells were evaluated. | Standard deviation | One way analysis of variance (One Way ANOVA) using mean values of mitochondrial inheritance of biological replicates collected in independent experiments with ad hoc normality (Shapiro-Wilk) and equal variance (Brown-Forsythe) tests, and post hoc Holm-Sidak test with all pairwise multiple comparisons |
| Fig 6B | Mean values of three biological replicates (n=3) based on three independent experiments (N=3); for each replicate 100 cells were evaluated. | Standard deviation | One way analysis of variance (One Way ANOVA) using mean values of biological replicates collected in independent experiments with ad hoc normality (Shapiro-Wilk) and equal variance (Brown-Forsythe) tests, and post hoc Holm-Sidak test with all pairwise multiple comparisons |
| Fig 6C | Mean values of three biological replicates (n=3) based on three independent experiments (N=3); for each replicate 100 cells were evaluated. | Standard deviation | Unpaired two-tailed Student's t-test with ad hoc normality (Shapiro-Wilk) and equal variance (Brown-Forsythe) tests using biological replicates collected in independent experiments |
| Fig 6E | Mean values of three biological replicates (n=3) based on three independent experiments (N=3); for each replicate 100 cells were evaluated. | Standard deviation | One way analysis of variance (One Way ANOVA) using mean values of biological replicates with ad hoc normality (Shapiro-Wilk) and equal variance (Brown-Forsythe) tests, and post hoc Holm-Sidak test with all pairwise multiple comparisons |
| Fig 7B | Mean values of three biological replicates (n=3) based on three independent experiments (N=3); for each replicate at least 100 cells were evaluated. | Standard deviation | One way analysis of variance (One Way ANOVA) using mean values of biological replicates collected in independent experiments with ad hoc normality (Shapiro-Wilk) and equal variance (Brown-Forsythe) tests, and post hoc Holm-Sidak test with all pairwise multiple comparisons |
| Fig 7F | Mean values of three biological replicates (n=3) based on three independent experiments (N=3); for each replicate 100 cells were evaluated. | Standard deviation | One way analysis of variance (One Way ANOVA) using mean values of biological replicates collected in independent experiments with ad hoc normality (Shapiro-Wilk) and equal variance (Brown-Forsythe) tests, and post hoc Holm-Sidak test with all pairwise multiple comparisons |
| Fig 8C | Mean values of three independent experiments (N=3); for each experiment 50 zygotes were evaluated. | Standard deviation | Unpaired two-tailed Welch's t-test with ad hoc normality (Shapiro-Wilk) test using mean values of independent experiments |

| Fig | Data | Error bars | Statistics |
|---|---|---|---|
| Fig 9C | Mean values of six independent experiments for Δ*dnm1* Δ*fzo1* (N=6) and three independent experiments for Δ*dnm1* Δ*fzo1* Δ*ypt11* and Δ*dnm1* Δ*fzo1* Δ*mmr1* (N=3); for each experiment at least 50 diploid cells were evaluated. | Standard deviation | One way analysis of variance (One Way ANOVA) using mean values of independent experiments with ad hoc normality (Shapiro-Wilk) and equal variance (Brown-Forsythe) tests, and post hoc Holm-Sidak test with all pairwise multiple comparisons |
