# Supplementary material for: Selective retention of dysfunctional mitochondria during asymmetric cell division in yeast
Source: PLoS Biol. 2023 Sep 18;21(9):e3002310. doi: 10.1371/journal.pbio.3002310 (PMC10538663; doi:10.1371/journal.pbio.3002310)
Supplement: S1 Raw images — (PDF) [file pbio.3002310.s018.pdf]

Original images for Fig 6F

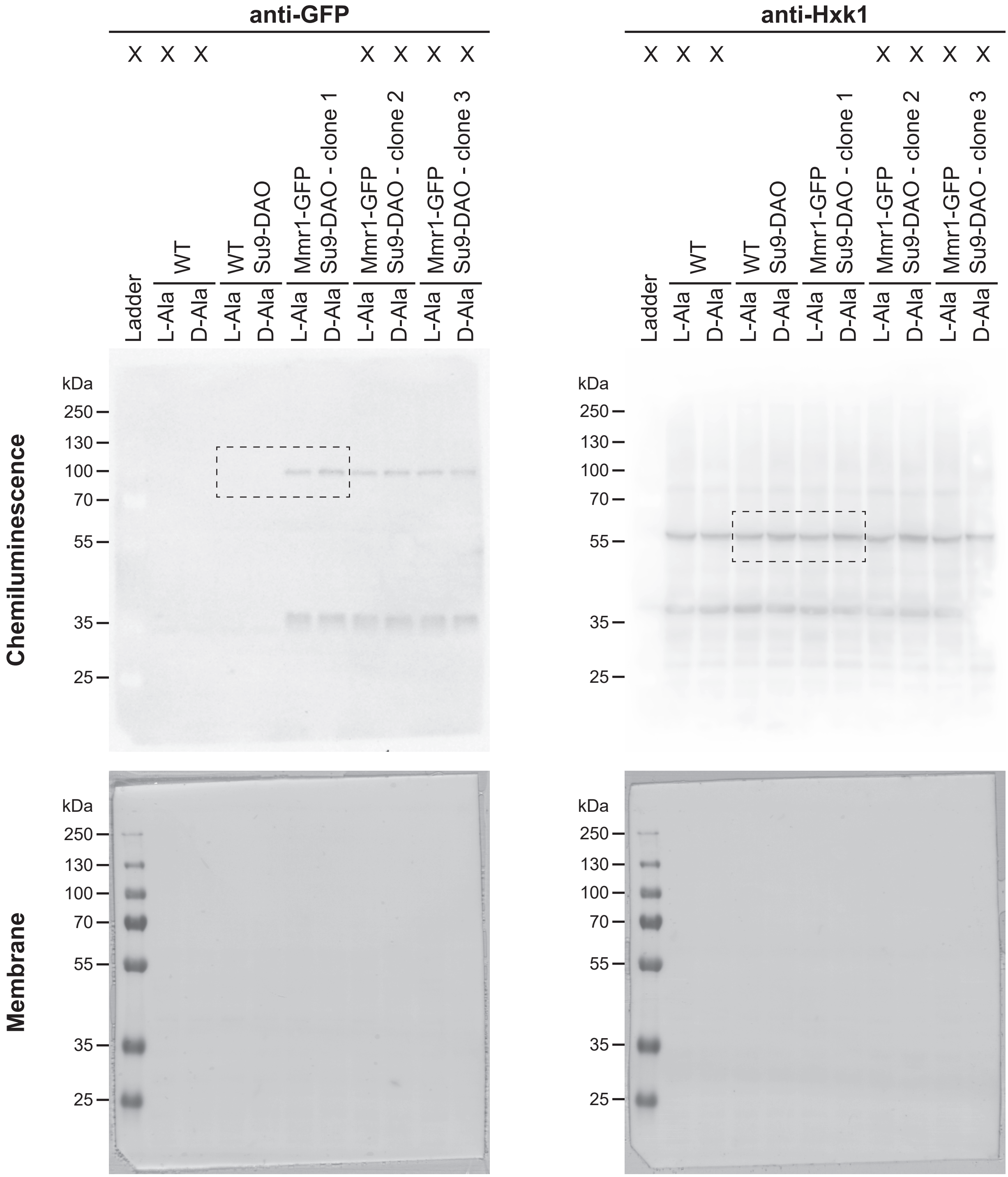

In both cases images of the same membrane are shown. The membrane was first probed with anti-GFP antibodies and subsequenty probed with anti-Hxk1 antibodies.
Images recorded with: Amersham ImageQuant 800, lower tray position, 3x3 binning
