# Supplementary material for: Selective retention of dysfunctional mitochondria during asymmetric cell division in yeast
Source: PLoS Biol. 2023 Sep 18;21(9):e3002310. doi: 10.1371/journal.pbio.3002310 (PMC10538663; doi:10.1371/journal.pbio.3002310)
Supplement: Attachment — Submitted filename: Response to Reviewers.pdf [file pbio.3002310.s019.pdf]

PBIOLOGY-D-23-00806R1

Point-by-point response to reviewers' comments

We are grateful to the reviewers for their thoughtful comments that helped us to further improve our manuscript. We are very happy to see that all three reviewers found our study interesting and well done. In the following, we describe how we addressed their specific points. In addition, we have added a source data file specifying all quantifications shown in the figures and formatted the manuscript according to the requirements of PLoS Biology. All changes to the text are highlighted in yellow in the 'track changes' version of the manuscript.

Reviewer #1

In the manuscript entitled "Selective retention of dysfunctional mitochondria...", Westermann and co-workers report the establishment of a method to selectively induce ROS formation in yeast mitochondria and use this tool to study the consequences that the resulting damage has on mitochondrial inheritance. They show that damage causes the fragmentation and retention of the affected mitochondria in the yeast mother cell, but not of unaffected mitochondria in the same cell, and dissect the mechanisms involved in decoupling the damaged mitochondria from the inheritance machinery. They show that especially the type V myosin/Myo2 receptor Mmr1 dissociates from damaged mitochondria, probably preventing the recruitment of Myo2, and hence stopping the motility of these organelles and their movement towards the bud.

Overall, this is a beautiful study for several reasons. First, the notion that "unfit" mitochondria are prevented from being inherited by the bud has been around for a while but the lack of appropriate assays and tools to study this idea rigorously had prevented from substantiating and confirming the idea in a definitive manner. Second, given these experimental limitations it had remained extremely difficult to acquire any mechanistic insights into how this may function. The manuscript of Westermann and colleagues addresses these two points in a very rigorous and definitive manner, establishing a very robust assay and using it to identify the key players involved. The data is very solid, convincingly conclusive and the experiments are extremely well controlled. The text is clear and reads well. The insights gained are very interesting and important. Therefore, I enthusiastically recommend publishing this very elegant study in your columns.

I see only one element that the authors might want to address before publication: The authors report that mitochondrial defects affect also vacuole inheritance (Figure 3G). However, the picture shown in that panel do not convey that message. Out of four cells shown in D-Ala, three show proper vacuolar inheritance. Interestingly, the affected cell does not show mitochondrial retention in the mother cell (mitochondrial signal is clearly seen in the bud). Perhaps, the authors could comment or show more representative pictures?

We thank the reviewer for these very positive comments. We have re-evaluated our data to see whether the inheritance of vacuoles and mitochondria under ROS stress is coupled or mutually exclusive. However, we did not find any significant interdependence, neither positive nor negative. We exchanged one of the images for a more representative one (i.e. a cell that shows inheritance of peroxisomes only).

Reviewer #2

In the manuscript by Chelius et al, the authors describe an investigation into the mechanism of selective mitochondrial retention during budding and mitosis in S. cerevisiae. They first demonstrate that yeast mitochondria respond to H2O2 by fragmentation, as previously reported by others, and by

loss of motility due to loss of actin cable structures. To separate mitochondria-specific responses from indirect effects on other cellular structures the authors express mitochondrially targeted D-amino acid oxidase. They demonstrate that mitochondrially targeted DAO causes mitochondrial dysfunction and morphological fragmentation in response to D-amino acid supplementation. They demonstrate that this is independent of oxphos activity and show that the fragmentation response is a result of inhibition of mitochondrial fusion. In addition, this also inhibited mitochondrial transmission to the bud during mitosis. These appear to be mitochondria-specific as actin cables remained intact and myo2 localization was not affected. The authors then ask whether the fragmentation phenotype is linked to the bud transport phenotype, by testing whether it is mitochondrial size that affects bud transport, or dynamics. They find that they can uncouple the inheritance phenotype from the fragmentation phenotype, by using double dnm1D fzo1D cells. Importantly they demonstrate that increasing Myo2 attachment to mitochondria by over expression of Ypt11 or Mmr1 can suppress the mitochondrial inheritance defect of mitochondrial DAO activity. Somewhat paradoxically, deletion of MMR1, but nor YPT11, also rendered mitochondrial inheritance independent of DAO expression. In agreement with a role for these proteins in regulating Myo2 association with mitochondria, an artificially mitochondrial-tethered Myo2 construct also rendered cells DAO- insensitive.

The authors then carry out a series of experiments in artificially heteroplasmic diploids containing separate noninteracting pools of DAO positive and DAO negative mitochondria showing that cells can select the DAO negative mitochondria for inheritance, and that Mmr1 is required for this selection.

Overall, this is an elegant paper that contains well thought out experiments and sheds new light on the enigmatic process of selective mitochondrial inheritance. Nonetheless, I find a number of weaknesses that I suggest the authors address before considering publication.

We are grateful to the reviewer for these positive remarks and his/her thoughtful suggestions.

First, the authors are using the mt-DAO construct to selectively generate H2O2 in mitochondria. This is an elegant approach, which seems to indicate that the target for H2O2 action is mitochondrial, and suggests some kind of signaling mechanism that would then regulate the cytoplasmic factors such as Ypt11/Mmr1. However the authors do not rule out the possibility that mitochondrial H2O2 is simply leaking out and directly activating a cytoplasmic response. The authors could address this by asking whether overexpression of cytosolic versus mitochondrial catalase can suppress the effects of DAO.

The reviewer addresses a key point of our study. We show that stressed and non-stressed mitochondria that share the same cytosol in the same cell behave remarkably differently (Figs. 8 and 9, Videos 7 and 9). If the activity of Su9-DAO would activate a cytosolic response, e.g. by H_2O_2 leaking out, we would have expected that all mitochondria that are present in the same cell would react in a similar manner, which is clearly not the case. To make this point more clear, we have added the information that even mitochondria that are in very close proximity behave differently (lines 372-376). Please see also lines 350-356 and 403-407 that refer to this important point. In agreement with the editors' decision letter, we did not perform additional experiments to address this point.

Close contacts between stressed and non-stressed mitochondria can also be seen in the figure shown below. Δ*dnm1* Δ*fzo1* cells expressing Su9-DAO were treated with D-alanine as in Figure 9. 49 randomly chosen cells are shown, most of which contain stressed mitochondria located right next to non-stressed organelles. These images are from a preliminary data set. As we did not examine the location of mitochondria relative to each other in a systematic manner, we prefer not to show this experiment in the manuscript.

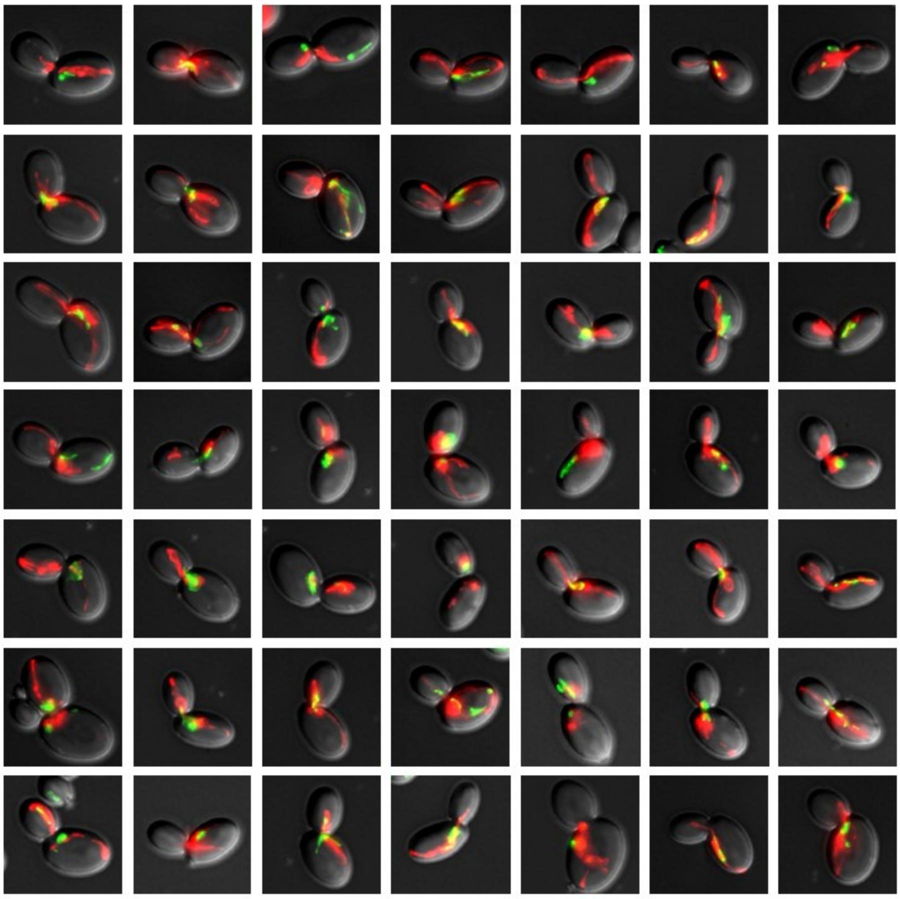

A second weakness is that I see, is that overexpression of Mmr1 suppresses the effects of mtDAO, but so does the deletion of Mmr1! I think the authors must address this 'paradox', whether experimentally or in the discussion.

We thank the reviewer for this point. We agree that it is not intuitively comprehensible that deletion and overexpression of *MMR1* have the same effect. We propose that this is the result of a complex regulatory network that controls mitochondrial inheritance. This is now discussed in lines 441-452.

Reviewer #3

In this manuscript the authors have studied the impact of oxidative damage originating in mitochondria on mitochondrial inheritance during cell division in yeast. This was performed by expressing D-amino oxidase in mitochondrial matrix, and incubation of cells with the substrate D-alanine or L-alanine as a control. The study is interesting and the most relevant observations are: a) mitochondrial oxidative stress causes specific alterations of the affected mitochondria, and not of the normal mitochondrial present in the same cell, and b) oxidative stress causes the dislocalization of Mmr1 in ROS-stressed mitochondria. The manuscript shows a number of weak aspects that require some attention.

We thank the reviewer for his/her assessment that this is an interesting study and for the valuable suggestions that helped us to further improve the manuscript.

1. The 10-fold serial dilution experiments shown in the manuscript should be quantified (such as in figures 1A and 1G). Why L-alanine impacts on cells grown in glycerol plus ethanol? Please, provide an explanation.

The serial dilutions of yeast cells, as shown in Figures 2A, G, 4C, and 7D, represent the standard assay in yeast genetics. We have reproduced these experiments several times and show one representative example. A quantification is not common practice and we feel that it would not yield any added value.

The reviewer is correct in noting that cells grown on SCGE plates containing 20 mM L-Ala grow slightly slower than on plates lacking L-Ala. This effect is reproducible, however, the reason for this is not clear. Importantly, the control strain demonstrates that this effect is independent of the presence of Su9-DAO. Please note that all experiments addressing mitochondrial inheritance were performed in liquid medium containing fermentable carbon sources, SCD or SCGal, i.e. conditions that did not show this effect.

2. During the manuscript the authors indicate that mitochondrially produced ROS enhances the percentage of cells that do not inherit any mitochondria during division. This is mentioned in figure 1B but in others. The authors should provide an explanation for that and specifically refer to this in the rest of the figures.

Fig. 1B shows mitochondrial morphology changes upon exogenously added H_2O_2. We assume that the reviewer's comment refers to Fig. 3B. This is the first time we describe the effect of mitochondrially produced ROS on mitochondrial inheritance; i.e. we find cells showing efficient inheritance, reduced inheritance, and no inheritance of mitochondria. The same information is also contained in the subsequent Figures 4B, E, 5B, D, F, 6B, 7B, F: The bar diagrams show efficient inheritance (black) and reduced inheritance (grey). The difference of the sum of these categories and 100% is the percentage of cells showing no mitochondrial inheritance. We choose this kind of representation of our data in order not to overload the figures. We have added an explanatory sentence to the legend of Fig. 4B. All data, including the numbers of buds devoid of mitochondria, are now also available in the newly added source data file (S1 Datasheet).

3. In Figure 4, the authors should document the impact of just depletion of Fzo1 on mitochondrial inheritance. Otherwise they cannot conclude that the effects of ROS production are independent of mitochondrial fusion.

We have previously shown that deletion of *FZO1* causes a significant mitochondrial inheritance defect because of mitochondrial fragmentation (Böckler et al. 2017, which is reference #61 in the present manuscript). In the experiment shown in Figure 4 we wanted to analyze the behavior of mitochondria that are unable to undergo fragmentation. Therefore, we analyzed Δ*dnm1*, a mutant containing interconnected mitochondrial networks that are unable to fragment because of a block of mitochondrial fission, and Δ*dnm1* Δ*fzo1*, a mutant that has wild type-like mitochondria that are no longer dynamic, i.e. defective in both fusion and fission. From these experiments we conclude that impaired inheritance of ROS-stressed mitochondria is not caused by mitochondrial fragmentation, i.e. an activity that is dependent on mitochondrial fission rather than fusion. We changed the headings of the results section and figure legend (which might have been misleading) and rewrote the text (lines 210-229) to make this more clear. Please note that in addition to the Δ*dnm1* Δ*fzo1* mutant shown in Figure 4A-C also the experiments shown in Figures 2H, 8, and 9 demonstrate that mitochondrial fusion is not required for preferential inheritance of non-stressed mitochondria.

Nevertheless, we analyzed the effect of Su9-DAO in Δ*fzo1*. Consistent with the results mentioned above, we do find a mitochondrial inheritance defect upon mitochondrial ROS production (D-Ala) in comparison to control conditions (L-Ala), as shown in the Figure below. However, the Δ*fzo1* strain shows a very strong mitochondrial inheritance defect, even under control conditions, and the behavior of mitochondria and quantification of mitochondrial inheritance is not comparable to the

wild type when mitochondria are already highly fragmented to begin with. Therefore, we decided not to include these data in the manuscript.

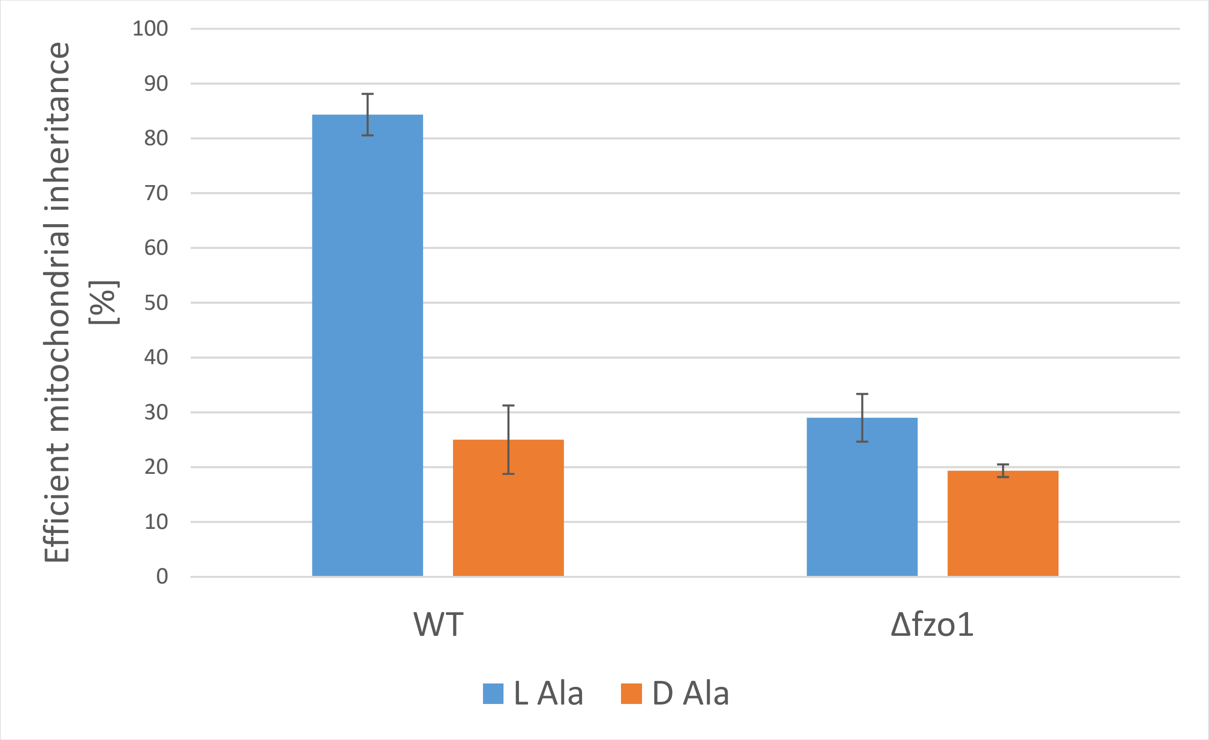

Mitochondrial inheritance in wild type and Δfzo1 strains expressing Su9-DAO was analyzed as in Figure 4 A and B. Bars show the percentage of budding cells with efficient mitochondrial inheritance. Error bars are SD of three biological replicates.

4. Data from Figure 6 suggest that Mmr1 disappear from the outer mitochondrial membrane of those mitochondria producing ROS. Is that due to degradation of the protein or is a consequence of dissociation of Mmr1 from mitochondria? Do you see alterations in the total content of Mmr1 in cells upon ROS production? This is relevant in order to understand the process that takes place under ROS conditions, and also based on data by Obara et al indicating an active proteolysis of Mmr1 protein (PMID: 35422486).

We thank the reviewer for this valuable suggestion and performed the proposed experiment. We observed that ROS stress does not induce degradation of Mmr1-GFP, as is evident from Western blots of total cell extracts. This new experiment is shown in Fig. 6F. Reference [97] has been added for the method used for protein extraction from yeast cells.

5. A basic question not addressed in the manuscript is whether there are differences in mitochondrial respiration between daughter and mother cells under conditions of high mitochondrial ROS production. This missing piece of evidence would validate the concept of a retention of damaged mitochondria in mother cells, and it would enhance the message of the manuscript.

We agree with the reviewer that it would be extremely interesting to visualize respiratory activity of mitochondria in buds and mother cells of ROS-stressed cells. However, this is technically not feasible, at least not with the assays that we are aware of. In agreement with the editors' decision letter, we did not perform additional experiments to address this point.
